# Evaluating the Reliability of Neurological Pupillary Index as a Prognostic Measurement of Neurological Function in Critical Care Patients

**DOI:** 10.7759/cureus.28901

**Published:** 2022-09-07

**Authors:** Muhammad S Ghauri, Arisa Ueno, Sumayya Mohammed, Dan E Miulli, Javed Siddiqi

**Affiliations:** 1 Neurosurgery, California University of Science and Medicine, Colton, USA; 2 Neurology, Arrowhead Regional Medical Center, Colton, USA; 3 Neurosurgery, Arrowhead Regional Medical Center, Colton, USA; 4 Neurosurgery, Desert Regional Medical Center, Palm Springs, USA; 5 Neurosurgery, Riverside University Health System Medical Center, Moreno Valley, USA

**Keywords:** neurological examination, pupillary reflex, neurosurgery, intensive care, neurocritical care, critical care

## Abstract

Background

Neurological pupil index (NPi) is a novel method of assessing pupillary size and reactivity using pupillometry to reduce human subjectivity. This paper aims to evaluate the use of NPi as a potential prognostic tool in a broad population of neurocritical care patients by observing the correlation between NPi, modified Rankin Scale (mRS), and Glasgow Coma Scale (GCS).

Methods

Our data was collected from 194 patients in the neurosurgical intensive care unit (ICU) at Arrowhead Regional Medical Center (ARMC), as determined by the power calculation. We utilized the Kolmogorov-Smirnova and Shapiro-Wilk normality tests with Lilliefors significance correction. Pearson product-moment correlation was performed between average final NPi and final GCS. Multi-variate linear regression and analysis of variance (ANOVA) were used to evaluate the association and predictive capabilities of NPi on GCS and discharge mRS. Finally, we evaluated whether age, ethnicity, sex, length of stay (LOS), or discharge location were significantly associated with NPi.

Results

We observed a significant correlation between final GCS and NPi (r=0.609, p<0.001). Our regression analysis revealed that NPi significantly predicted GCS and mRS scores; however, no associations were found between age, ethnicity, sex, LOS, or discharge location. Limitations of our study include a single institutional study with a lack of disease subtyping and the inability to quantify the predictive ability of NPi.

Conclusion

The analysis revealed a strong correlation between final GCS and average final NPi. NPi was also able to significantly predict GCS and mRS scores. The correlation between NPi and established methods to determine neurological function, such as mRS and GCS, suggests that NPi can be a good prognostication tool for neurological diseases.

## Introduction

Pupillary size and reactivity are among the major non-invasive methods of assessing neurological function. During the pupillary reflex exam, the pupil's size and symmetry are measured, and the rate of reactivity is classified as either brisk, sluggish, or non-reactive. Abnormal measurements can indicate diseases such as stroke, tumors, and traumatic brain injuries [1-3]. It is a valuable prognostic tool for assessing the patient's neurological health [4]. Current methods of assessing pupillary reflex include the Glasgow Coma Scale (GCS) and modified Rankin Scale (mRS). To date, many variations of these clinical tools have been developed to further refine the prognosticative ability of patient outcomes. However, these measurements are predisposed to inaccuracy due to subjectivity, inexperience, language barriers, iatrogenic barriers (e.g., intubation, sedation), and lack of standardization of the examiner [5-9]. Neurological pupil index (NPi) is a new practice established by NeuroOptics, Inc. (Irvine, USA) that measures the size, latency, and velocity parameters and quantifies them on a scale from zero to five, zero being non-reactive, and a score equal to or above three indicating normal pupil behavior [6]. NPi utilizes automated pupillometry to decrease human subjectivity and minimize administration time to increase the efficiency and accuracy of neurological assessments. Evaluating the change in NPi could potentially serve as a more robust prognostication tool to assess the recovery of the brain in neurocritical care.

With the development of the international curing coma campaign (COME TOGETHER), we have seen increasing interest to improve the assessment of patients with impaired neurological function [7-10]. Previous studies have investigated the utility of these prognostic measurements in combination and even integrated in prognostic modeling calculators, such as the international mission for prognosis and clinical trials in traumatic brain injury (IMPACT) and corticoid randomization after significant head injury (CRASH) [8,11-13]. These studies found that among a variety of these clinical measurements, GCS, NPi, and mRS were all significant predictors of patient outcome in the context of traumatic brain injury (TBI) [14-17]. Other studies have corroborated these findings, but many of these analyses hone into specific clinical contexts, such as TBI and stroke [18-20]. More studies are needed to compare prognostic capabilities across a broad range of neurocritical diseases to increase the generalizability and feasibility of automated pupillometry.

If we can establish a correlation between NPi, mRS, and GCS, it could support the reliability of NPi as an alternative and potentially more robust prognostic tool in critical care patients. In this study, we used a pupillometer to measure the NPi in 194 subjects in the neurosurgical intensive care unit (ICU) at Arrowhead Regional Medical Center (ARMC) in San Bernardino, California. We hypothesized that NPi and GCS would be positively correlated, mRS and NPi would be negatively correlated, and NPi could significantly predict GCS and mRS scores. Therefore, NPi can be used similarly to or in conjunction with GCS as a tool to assess neurologic function across a varied neurocritical patient population.

## Materials and methods

We collected data from patients in the neuro-ICU at ARMC. The following demographic and clinical information were obtained from medical records to describe patient baseline characteristics: age, sex, ethnicity, length of stay (LOS), and discharge disposition/location. Clinical neurological assessments (i.e., GCS, mRS) were obtained using conventional established methods upon admission and at the time of discharge. NPi measurements were obtained at admission, every four hours (every hour for critically unstable patients), and at ICU discharge using the NeuroOptics Pupillometer (Irvine, CA, USA) version 2.00. NPi values greater than 3.0 were characterized as normal, and NPi values less than 3.0 was considered abnormal.

Initially, we conducted a pre-study power calculation using G*Power (version 3.1.9.7; Heinrich Heine University Düsseldorf, Germany) to compute the statistically significant sample size needed for data collection. Our a priori analyses suggested a sample size of 191 patients to achieve a power of 0.80. Next, we evaluated the homogeneity of our data distribution by using the Kolmogorov-Smirnova and Shapiro-Wilk normality tests with Lilliefors significance correction. We then performed Pearson product-moment correlation analyses to identify any statistically significant correlations between our continuous variables average final NPi, Final GCS, and LOS. The obtained Pearson correlation coefficients were categorized as weak (0.00-0.30), moderate (0.31-0.60), and strong (>0.60). The association of NPi, GCS, and mRS and the ability of NPi to predict GCS and mRS, was determined using multiple and ordinal regression analysis. Finally, we determined whether several predictor variables, such as age, ethnicity, sex, LOS, and discharge location, could significantly predict a patient's NPi score using multivariate regression and ANOVA. All statistical analyses were performed using SPSS statistics software V28.0.1.0 (IBM Inc., Armonk, USA).

We conducted this study in compliance with the principles of the Declaration of Helsinki. The study's protocol was reviewed and approved by the Institutional Review Board of Arrowhead Regional Medical Center (#22-21). Informed consent was waived.

Participants of the study were those that were admitted to the neuro ICU at ARMC. Their sex was determined through medical records. This study did not involve an exclusive population. Ethnicity was self-determined by patients upon initial admission to the hospital and was included in this study to determine any patterns between NPi and prognosis in certain groups.

## Results

Patient characteristics/demographics were obtained at baseline (Table 1). Categorical variables (age, sex, ethnicity, discharge location) were reported as frequencies and percentages. Continuous variables (initial/final/average GCS, NPi, mRS, LOS) were described as mean and standard deviation. One hundred and ninety-three patients were included in this study. Most of the patients were males (n=111, 57.5%) and Hispanic (n=117, 60.6%). The mean (±SD) age was 62.44±14 years, and LOS was 7.25±9.21 days. The majority of patients were discharged home (n=121, 62.69%), 27 patients (14%) to rehabilitation centers (REHAB), 20 patients (10.36%) to skilled nursing facilities (SNF), seven patients (3.63%) to "inpatient to outside hospital" (INPT to OSH), five patients (2.6%) to "leave against medical advice" (AMA), two patients (1.04%) to hospice, one patient (0.52%) to long term acute care (LTAC), and three out of 193 patients expired (1.55%, average final NPi=4.24).

**Table 1:** Patient demographic characteristics Categorical variables are presented as frequency (%); continuous variables are presented as mean±SD. The total may not sum to 100% because of rounding. AMA - left against medical advice; INPT to OSH - inpatient to outside hospital; LTAC - long-term acute care; REHAB - rehabilitation; SNF - skilled nursing facility; Avg - average; mRS - modified Rankin Scale; NPi - neurological pupil index; GCS - Glasgow Coma Scale

Variable	Value (n=193)
Age	62.4±14
Sex	
Male	111 (57.5%)
Female	82 (42.5%)
Ethnicity	
Asian	11 (5.7%)
African American	30 (15.5%)
Caucasian	30 (15.5%)
Hispanic	117 (60.6%)
Pacific Islander	1 (0.5%)
Other	2 (0.5%)
Unknown	2 (1%)
Initial GCS	14.3±1.6
Final GCS	14.4±1.6
Delta GCS	0.1±0.9
Initial NPi	
Left	4.4±0.6
Right	4.4±0.5
Avg	4.4±0.2
Final NPi	
Left	4.4±0.7
Right	4.3±0.6
Avg	4.4±0.3
Delta NPi	
Left	-4.00E-04
Right	-2.30E-02
Avg	-1.2E^-2 ^±0.3
Initial mRS	2.4±1.6
Final mRS	2.4±1.7
Delta mRS	5.2E^-3^±1.0
Length of stay (LOS)	7.3±9.2
Discharge location	
AMA	5 (2.6%)
Expired	3 (1.6%)
Home	121 (62.7%)
Hospice	2 (1.0%)
IPT to OSH	7 (3.6%)
LTAC	1 (0.52%)
REHAB	27 (14%)
SNF	20 (10.4%)

NPi, GCS, and mRS measurements were taken upon admission (initial NPi) and at the time of discharge (final NPi). NPi measurements at each time point were measured individually in each eye (left and right), then averaged. Furthermore, the change in NPi (delta NPi) was measured by taking the difference in final vs initial NPi in each eye, then averaged across both eyes (average delta NPi). The mean ± SD of initial GCS was 14.28±1.61, final GCS was 14.38±1.59, delta GCS was 0.098±0.98, initial mRS was 2.36±1.62, final mRS was 2.35±1.72, delta mRS was 0.0052±1.03. The average initial NPi was 4.36±0.22 (left=4.36±0.61, right=4.37±0.51), average final NPi was 4.35±0.32 (left=4.36±0.66, right=4.34±0.59), average delta NPi was -0.0118±0.28 (left=-0.0004, right=-0.0233).

Our statistical analysis revealed a homogeneous distribution of data by age, sex, ethnicity, final GCS, average final NPI, final mRS, LOS, and discharge location. Table 2 summarizes the correlation coefficients obtained from our statistical models. We identified a significant correlation between NPi, GCS (r=0.609, p<0.001), and mRS (r=-0.495, p<0.001). Regression analysis was also used to test if NPi could significantly predict GCS, and discharge mRS score. The overall regression analyses were statistically significant for GCS (R^2^=0.37, F(331,561)=112.01, p≤0.001) and discharge mRS (R^2^=0.241, F(213, 657) = 112.01, p≤0.001). Table 3 outlines the results of our multiple regression and ANOVA models used to identify associations between NPi and predictors of age, sex, ethnicity, LOS, and discharge location. Although our model was statistically significant, when looking at the unique individual contribution of our predictors, we found that age, ethnicity, sex, LOS, and discharge location (DL) displayed no significant associations or ability to predict NPi.

**Table 2:** Correlation coefficients between patient demographic characteristics and clinical measurements Strong correlation (r≥0.60); moderate correlation (r=0.31-0.60); weak correlation (r=0.00-0.30) **Correlation is significant at the p=0.01 level (2- tailed) *Correlation is significant at the p=0.05 level (2- tailed) GCS - Glasgow Coma Scale; mRS - modified Rankin Scale; LOS - length of stay; mRS - modified Rankin Scale; NPi - neurological pupil index

	Average Final NPi	Final GCS	Discharge mRS	LOS	Discharge location	Sex	Ethnicity	Age
Average final NPi	1	0.609**	-0.495**	-0.082	-0.062	-0.105	-0.053	0.080
Final GCS	0.609**	1	-0.724**	-0.175	-0.135	-0.112	0	-0.176*
Discharge mRS	-.0495**	-0.724**	1	0.283**	0.454**	0.212**	0.001	0.249**
LOS	-0.082	-0.175*	0.283**	1	0.410**	0.212**	0.001	0.249**
Discharge location	0.062	0.135	0.454**	0.410**	1	0.108	0.090	0.073
Sex	0.105	-0.112	0.212**	-0.092	0.108	1	-0.001	0.062
Ethnicity	-0.053	0.000	0.001	-0.160*	0.090	-0.001	1	0.014
Age	0.080	-0.176*	0.249**	0.042	0.073	0.062	0.014	1

**Table 3:** Multiple regression of predictor variables of NPi GCS - Glasgow Coma Scale; mRS - modified Rankin Scale; LOS - length of stay; DL - discharge location; AMA - Left against medical advice; INPT to OSH - inpatient to outside hospital; LTAC - long-term acute care; REHAB - rehabilitation; SNF - skilled nursing facility; NPi - neurological pupil index

Variables	B	β	R^2^	t	Sig	95% CI	
GCS	1.895	0.609	0.379	10.584	<0.001	1.542	2.248
mRS	-1.52	-0.495	0.245	-7.846	<0.001	-1.904	-1.139
LOS	-0.006	-0.081	0.007	-1.143	0.255	-0.017	0.005
Age	0.004	0.08	0.006	1.108	0.269	-0.003	0.011
DL		-0.062	0.149		0.021		
AMA	0.221	0.051		0.7		-0.401	0.842
Expired	-2.049	-0.367		-4.128		-3.029	-1.069
Home	-0.162	-0.113		-0.523		-0.773	0.449
Hospice	-0.495	-0.073		-0.866		-1.625	0.634
INPT to OSH	-0.123	-0.033		-0.309		-0.906	0.66
LTAC	0.07	0.007		0.093		-1.408	1.548
REHAB	-0.359	-0.18		-1.085		-1.011	0.294
SNF	-0.2	-0.088		-0.59		-0.871	0.47
Ethnicity		-0.053	0.011		0.915		
Hispanic	0.006	0.004		0.059		-0.196	0.208
Asian	-0.086	-0.029		-0.398		-0.512	0.34
Black	0.017	0.009		0.119		-0.266	0.301
Pacific Islander	0.268	0.028		0.398		-1.061	1.597
White	-0.024	-0.012		-0.167		-0.301	0.254
Unknown	0.217	0.032		0.453		-0.73	1.165
Other	-0.144	-0.021		-0.295		-1.104	0.816
Sex		-0.105	0.011		0.145		
Male	0.147	0.105		1.452		-0.053	0.346
Female	-0.147	-0.105		-1.462		-0.346	0.051

## Discussion

As we hypothesized, our results identified a strong correlation between average final NPI and final GCS scores. The correlation between these two variables suggests that NPi measurements could be used similarly to GCS as an effective predictor of prognosis. Our subsequent regression model reveals that our utilization of these three measurements is significantly correlated within varying disease contexts present in our patient population. Since GCS and discharge mRS have been shown to effectively predict patient prognosis, NPi's ability to significantly predict these two measurements supports the possibility of using NPi as an alternative predictor of prognosis [21-22]. However, further multi-institutional studies are needed to evaluate the potential superiority of NPi as a predictor of the prognosis within a variety of neurological disease contexts.

Interestingly, although NPi, GCS, mRS displayed significant relationships, we found that only NPi was able to significantly predict discharge location. Although the majority of patients were discharged home, being able to identify relationships between a patient's "measured" neurological health and inevitable discharge location could help improve hospital resource utilization and care management. This highlights the need to explore whether these trends can be seen at other institutions and if better categorizations are needed to better identify significant relationships and predictive capabilities within these variables.

Our study also looked at whether demographic parameters such as age, sex, and ethnicity played a role in predicting our patients' prognoses, as measured by NPi. Overall, we failed to identify any significant associations between these variables, which could be attributed to our predominantly Hispanic patient population. Finding strong correlations between certain demographic groups and the prognostic capabilities of NPi, GCS, and mRS could help make better-informed decisions. However, such strong demographic patterns could also question the external validity of these measurement tools in a varied patient population. These results convey that NPi and GCS can be similarly used in predicting prognosis among a potentially diverse patient demographic, although more studies are needed to confirm these findings.

Our study does have some limitations. First, although our sample size displayed appropriate statistical power, a majority of our patients are Hispanic and over the age of 50. Further analysis is needed within a multi-institutional study with more patients to ensure reproducibility and generalizability to a diverse patient population. Second, since our study aimed to evaluate the reliability of NPi in all neurocritical care patients, we did not stratify our patients based on the clinical context. Hence, we are unable to take into account specific neurological diseases and the potential confounding effects on predicting patient prognosis. Finally, our correlation analyses cannot determine any causations or quantify how well these tools predict long-term patient outcomes and should be interpreted with these points in mind.

Overall, our study offers additional insight into how NPi, which could serve as a more objective and efficient method of evaluating patient prognosis, relates to conventional methodologies like GCS and mRS. Despite their widely accepted use, these tools are inherently slightly more subjective and more prone to potential differences in inter-observer reliability. Our future studies will aim to quantify how well NPi can predict long-term patient outcomes within specific disease subtypes. We plan to replicate this analysis as part of a multi-institutional study and corroborate NPi as a potentially superior prognostic tool for neurocritical care patients.

## Conclusions

This study examined the correlation between NPi and the prognosis of neuro ICU patients at ARMC in San Bernardino County, CA. Our results demonstrated a significant predictive association between average final NPi, final GCS, and discharge mRS scores. The relationship between these variables suggests that NPi measurements could be used similarly to GCS as an effective predictor of prognosis. Our results suggest that NPi is a promising tool in our armamentarium to gauge a patient's neurological health, monitor disease/treatment progression, and better predict prognosis in modern neurocritical practice.
